# Transcriptomic and small RNA response to Mayaro virus infection in *Anopheles stephensi* mosquitoes

**DOI:** 10.1371/journal.pntd.0010507

**Published:** 2022-06-28

**Authors:** Cory Henderson, Marco Brustolin, Shivanand Hegde, Gargi Dayama, Nelson Lau, Grant L. Hughes, Christina Bergey, Jason L. Rasgon

**Affiliations:** 1 Department of Entomology, The Pennsylvania State University, University Park, Pennsylvania, United States of America; 2 Department of Genetics, Rutgers University, New Brunswick, New Jersey, United States of America; 3 Departments of Vector Biology and Tropical Disease Biology, Centre for Neglected Tropical Disease, Liverpool School of Tropical Medicine, Liverpool, United Kingdom; 4 School of Medicine, Boston University, Boston, Massachusetts, United States of America; 5 Unit of Entomology, Department of Biomedical Sciences, Institute of Tropical Medicine, Antwerp, Belgium; University of Glasgow, UNITED KINGDOM

## Abstract

Mayaro virus (MAYV) is an arboviral pathogen in the genus *Alphavirus* that is circulating in South America with potential to spread to naïve regions. MAYV is also one of the few viruses with the ability to be transmitted by mosquitoes in the genus *Anopheles*, as well as the typical arboviral transmitting mosquitoes in the genus *Aedes*. Few studies have investigated the infection response of *Anopheles* mosquitoes. In this study we detail the transcriptomic and small RNA responses of *An*. *stephensi* to infection with MAYV via infectious bloodmeal at 2, 7, and 14 days post infection (dpi). 487 unique transcripts were significantly regulated, 78 putative novel miRNAs were identified, and an siRNA response is observed targeting the MAYV genome. Gene ontology analysis of transcripts regulated at each timepoint shows a number of proteases regulated at 2 and 7 dpi, potentially representative of Toll or melanization pathway activation, and repression of pathways related to autophagy and apoptosis at 14 dpi. These findings provide a basic understanding of the infection response of *An*. *stephensi* to MAYV and help to identify host factors which might be useful to target to inhibit viral replication in *Anopheles* mosquitoes.

## Author summary

Mayaro virus (MAYV) is a mosquito-borne Alphavirus responsible for outbreaks in South America and the Caribbean. In this study we infected *Anopheles stephensi* with MAYV and sequenced mRNA and small RNA to understand how MAYV infection impacts gene transcription and the expression of small RNAs in the mosquito vector. Genes involved with innate immunity and signaling pathways related to cell death are regulated in response to MAYV infection of *An*. *stephensi*, we also discovered novel miRNAs and describe the expression patterns of miRNAs, siRNAs, and piRNAs following bloodmeal ingestion. These results suggest that MAYV does induce a molecular response to infection in its mosquito vector species.

## Introduction

Mayaro virus (MAYV) is a mosquito-borne, enveloped positive-sense single-stranded RNA virus in the genus *Alphavirus*, first isolated from the blood of five febrile workers in Mayaro county, Trinidad in 1954 [1]. Symptoms of MAYV infection are similar to other arboviral infections such as dengue (DNV) or chikungunya viruses, (CHIKV) and include rash, fever, retro-orbital pain, headache, diarrhea, and arthralgia [2]. While no epidemics or outbreaks with MAYV being the causative agent have been recorded outside of South America, there have been imported cases reported in the Netherlands, Germany, France, and Switzerland [3–6], which demonstrates a need to understand the capacity for the virus to spread into naïve regions, such as the United States.

The principal mosquitoes transmitting MAYV naturally are thought to be the canopy-dwellers of the genus *Haemogogus*, maintaining the sylvatic cycle between non-human primates as primary hosts and birds as secondary hosts [7]. Human infections are sporadic due to the rare display of anthropophilic biting behaviors by *Haemogogus* mosquitoes, with transmission due to these species primarily occurring in rural regions with close proximity to forests [8]. Vector competence studies have identified anthropophilic and urban adapted species such as *Aedes aegypti* and *Ae*. *albopictus*, as well as the malaria parasite transmitters *Anopheles gambiae*, *An*. *stephensi*, *An*. *freeborni*, and *An*. *quadrimaculatus*, as being competent vectors for MAYV under laboratory conditions [9–12]. Transmission of an arbovirus with an anopheline mosquito as a primary vector is rare, only having been observed occurring regularly for o’nyong’nyong virus by *An*. *gambiae* and *An*. *funestus* in Uganda [13], with some limited evidence for CHIKV and Semliki Forest virus (SFV) [14].

As arboviral pathogens are transmitted between hosts primarily by arthropod vectors, transmission requires the virus to infect and disseminate from the midgut and salivary glands of the mosquito following an infectious bloodmeal [15]. Infection of salivary glands is required for arbovirus transmission. The midgut, however, presents the earliest stage of infection when a mosquito becomes presented with a viremic bloodmeal and if even a small fraction of the midgut is infected, the virus can become established and spread to the rest of the organism. The molecular underpinnings controlling why MAYV and these closely related viruses can infect and be transmitted by *Anopheles* is of epidemiological interest, yet remains poorly understood. A more complete understanding of this phenomenon requires investigation of the molecular pathways involved in viral infection of anopheline mosquitoes. Recent transcriptomic studies have identified a number of genes involved in classical immune pathways, RNA interference (RNAi), metabolism, energy production, and transport as being regulated in response to arboviral infection of mosquitoes [16–19]. In addition, studies focusing on small RNA identification and regulation have identified RNAi activity, such as miRNA, piRNA, and siRNA expression, in response to infection of mosquitoes by arboviruses [20–23].

The available evidence suggests that, should MAYV be introduced into a naïve region, outbreaks and epidemics of the resulting disease could be driven by anopheline vectors [9,24–25]. Aedine vectors are less likely to facilitate widespread transmission of MAYV due to the fact that *Aedes* were only observed to transmit the limited strain of MAYV, while anophelines could transmit both [9]. Because anopheline, and not aedine, mosquitoes could act as the primary transmitting vectors for MAYV, this study also provides an opportunity to understand how vector competence might emerge in this system and provide insight into why *Anopheles* are generally poor viral transmitters when compared to *Aedes* mosquitoes. We used RNA sequencing to study the transcriptomic and small RNA responses of *An*. *stephensi* to infection with MAYV via infectious bloodmeal at 2, 7, and 14 days post infection (dpi).

## Materials and methods

### *Anopheles stephensi* rearing

Protocols pertaining to mosquito rearing and presentation of infectious bloodmeal has been described elsewhere [9]. Briefly, *An*. *stephensi* (Liston strain) were provided by Johns Hopkins University (Baltimore, MD, USA). Mosquito colonies were reared and maintained at the Millennium Sciences Complex insectary (The Pennsylvania State University, University Park, PA, USA) at 27°C ± 1°C, 12 hour light 12 hour dark diurnal cycle at 80% relative humidity in 30×30×30-cm cages. Ground fish flakes (TetraMin, Melle, Germany) were used to feed larvae, and upon emergence adult mosquitoes were maintained with a 10% sucrose solution.

### Viral production and infection via bloodmeal

Mayaro virus strain BeAn 343102 (BEI Resources, Manassas, VA, USA) was utilized in this study, a genotype D strain originally isolated from a monkey in Para, Brazil, in May 1978. Virus-infected supernatant was aliquoted and stored at −80°C until used for mosquito infections. Viral stock titers were obtained by focus forming assay (FFA) technique. Adult female mosquitoes at 6 days post emergence that had not previously blood-fed were used for experimentation. Mosquitoes were allowed to feed on either human blood spiked with MAYV at 1*10^7^ FFU/mL or a control bloodmeal with no virus via a glass feeder jacketed with 37°C distilled water for 1 h.

At 2, 7, and 14 days post infection, mosquitoes were anesthetized with triethylamine (Sigma, St. Louis, MO, USA) and RNA was extracted from each individual mosquito using mirVana RNA extraction kit (Life Technologies) applying the protocol for extraction of total RNA. Infection was confirmed via qPCR using primers published by Wiggins et al. 2018 (Forward: 5′-TGGACCTTTGGCTCTTCTTATC-3′, Reverse: 5′-GACGCTCACTGCGACTAAA-3′) [10], a CT value of 20 or less was used to confirm infection (S1 Table). 3 pools of total RNA were created for each time point and infection status to be used for library preparation, each consisting of 750 ng of RNA from 4 mosquitoes for a total of 3 μg per pool as confirmed via nanodrop. The protocol for mosquito rearing, viral production, and infection via bloodmeal is described in more detail in Brustolin et al. 2018 [9].

### Transcriptomic library preparation and sequencing

All pools were sent to University of Texas Medical Branch for library preparation where total RNA was quantified using a Qubit fluorescent assay (Thermo Scientific) and RNA quality was assessed using an RNA 6000 chip on an Agilent 2100 Bioanalyzer (Agilent Technologies). See Etebari et al. 2017 for more detail on library preparation and sequencing [17]. 1 μg of total RNA per pool was poly-A selected and fragmented using divalent cations and heat (94°C, 8 min). The NEBNext Ultra II RNA library kit (New England Biolabs) was used for RNA-Seq library construction. Fragmented poly-A RNA samples were converted to cDNA by random primed synthesis using ProtoScript II reverse transcriptase (New England Biolabs). After second strand synthesis, the double-stranded DNAs were treated with T4 DNA polymerase, 5’ phosphorylated and then an adenine residue was added to the 3’ ends of the DNA. Adapters were then ligated to the ends of these target template DNAs. After ligation, the template DNAs were amplified (5–9 cycles) using primers specific to each of the non-complimentary sequences in the adapters. This created a library of DNA templates that have non-homologous 5’ and 3’ ends. A qPCR analysis was performed to determine the template concentration of each library. Reference standards cloned from a HeLa S3 RNA-Seq library were used in the qPCR analysis. Cluster formation was performed using 15.5–17 million templates per lane using the Illumina cBot v3 system. Sequencing by synthesis, paired end 75 base reads, was performed on an Illumina NextSeq 550 using a protocol recommended by the manufacturer.

### Small RNA library preparation and sequencing

Small RNA libraries were created using the New England Biolabs small RNA library protocol. See Saldaña et al. 2017 for more information on small RNA sequencing [21]. Library construction used a two-step ligation process to create templates compatible with Illumina based next generation sequence (NGS) analysis. Where appropriate, RNA samples were quantified using a Qubit fluorometric assay. RNA quality was assessed using a pico-RNA chip on an Agilent 2100 Bioanalyzer. Library creation uses a sequential addition of first a 3’ adapter sequence followed by a 5’ adapter sequence. A cDNA copy was then synthesized using ProtoScript reverse transcriptase and a primer complimentary to a segment of the 3’ adapter. Amplification of the template population was performed in 15 cycles (94°C for 30 sec; 62°C for 30 sec; 70°C for 30 sec) and the amplified templates were PAGE (polyacrylamide gel electrophoresis) purified (147 bp DNA) prior to sequencing. All NGS libraries were indexed. The final concentration of all NGS libraries was determined using a Qubit fluorometric assay and the DNA fragment size of each library was assessed using a DNA 1000 high sensitivity chip and an Agilent 2100 Bioanalyzer. Single end 75 base sequencing by synthesis on an Illumina NextSeq 550.

### Transcriptomic RNA sequencing data analysis

Raw sequencing data was uploaded to the ICS-ACI high performance computing cluster at Pennsylvania State University to perform all computational analyses. Transcriptomic libraries had adapters trimmed and low-quality bases removed using Trimmomatic read trimming software with base settings [26]. Quality trimmed reads were aligned to the current build of the *An*. *stephensi* Indian strain genome in Vectorbase (AsteI2) using the STAR RNA sequencing aligner [27]. Reads less than 75 bp in length and with a mapping quality of less than 20 were dropped from the analysis, and read counts were calculated in R using the rSubread package [28], following which a principal components analysis was performed and differential expression conducted using a negative binomial GLM with the EdgeR package [29]. Contrasts considered in the GLM were infected against control at 2, 7, and 14 dpi, and differences between 2–7 dpi and 7–14 dpi for infected treatments corrected for the response from the control treatments between the same time points. Gene IDs that were differentially expressed with a log2FC value of +/- 1 and P value < 0.05 were uploaded to g:Profiler to run GO term overrepresentation analysis [30].

### Small RNA sequencing data analysis

Small RNA libraries had adapters trimmed using Trimmomatic and were subsequently passed into the miRDeep2 pipeline to identify novel and known miRNAs in all samples and determine expression of all known and novel miRNAs at each time point and treatment status [31,26]. Novel miRNAs with a miRDeep score of less than 3, a minimum free energy value of less than– 20, or a non-significant Randfold p-value were considered false IDs and excluded from further analysis. miRNA targets were identified in the AsteI2 genome using miRanda software package [32]. Differential expression of miRNAs in response to infection status and time point was conducted using a negative binomial GLM with the EdgeR package and contrasts as described for the transcriptomic analysis [29]. miRNAs which were differentially expressed with log2FC +/- 1 and P value < 0.05 had their miRanda genomic targets uploaded to g:Profiler to determine if any GO terms were overrepresented by transcripts potentially regulated by differentially expressed miRNAs [33]. Correlation between significantly regulated miRNAs and transcripts was determined by performing Kendall rank correlation on logFC values of significantly regulated miRNAs against significantly regulated transcripts. Correlations were considered valid with P value < 0.05 and tau > 0.03. piRNAs and siRNAs were isolated from the small RNA libraries by selecting all 18–24 nt reads (siRNA) and 24–35 nt (piRNA) reads from the trimmed datasets and filtering out all identified mature miRNAs, and those mapping to the MAYV NC_003417.1 genome were considered potential piRNAs or siRNAs. piRNA and siRNA alignment to the AsteI2 genome was performed using Bowtie RNA sequencing aligner within the MSRG pipeline [34]. Observation of fastQC output for Control Day 7 Replicate 1 small RNA sequencing revealed poor sequencing results, so this replicate was omitted from all analyses in the small RNA focused portions of this study [35].

## Results/discussion

### Transcriptome

#### RNA sequencing

**Fig 1:**
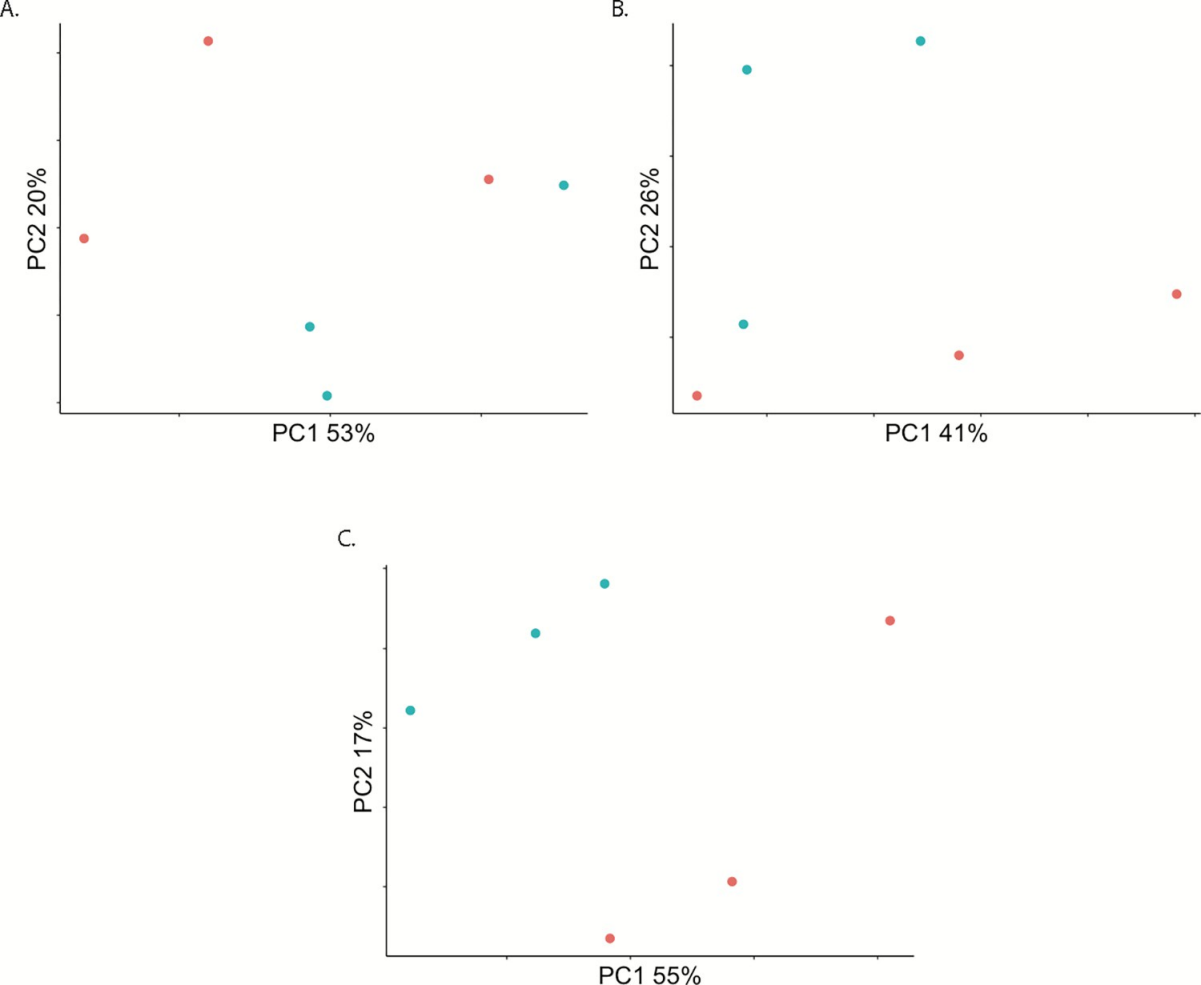
Principal Components Analysis (PCA) on filtered read counts mapping to annotated genes from the AsteI2 build of the *An*. *stephensi* genome in Vectorbase. A., B., and C. are read counts from samples in the 2, 7, and 14 dpi groupings respectively. In all PCAs, blue is Mayaro infected, and red are control.

We assayed genome-wide gene expression in pools of *An*. *stephensi* (Liston strain) experimentally infected with MAYV at 2, 7, and 14 dpi, along with blood fed uninfected negative controls. RNAseq libraries were sequenced on the Illumina NextSeq 550 platform, yielding 20.6–28.4 million paired end reads per library. (S1 Table). Principal components analysis (PCA) performed on read counts of each annotated gene in the *An*. *stephensi* (Indian strain) reference transcriptome (AsteI2) at each time point distributed infected and control samples into somewhat distinct groups and it is evident there is less diversity among infected samples than control in placement within PC space, however there is some overlap between infected and control (Fig 1).

#### Differential expression

**Fig 2:**
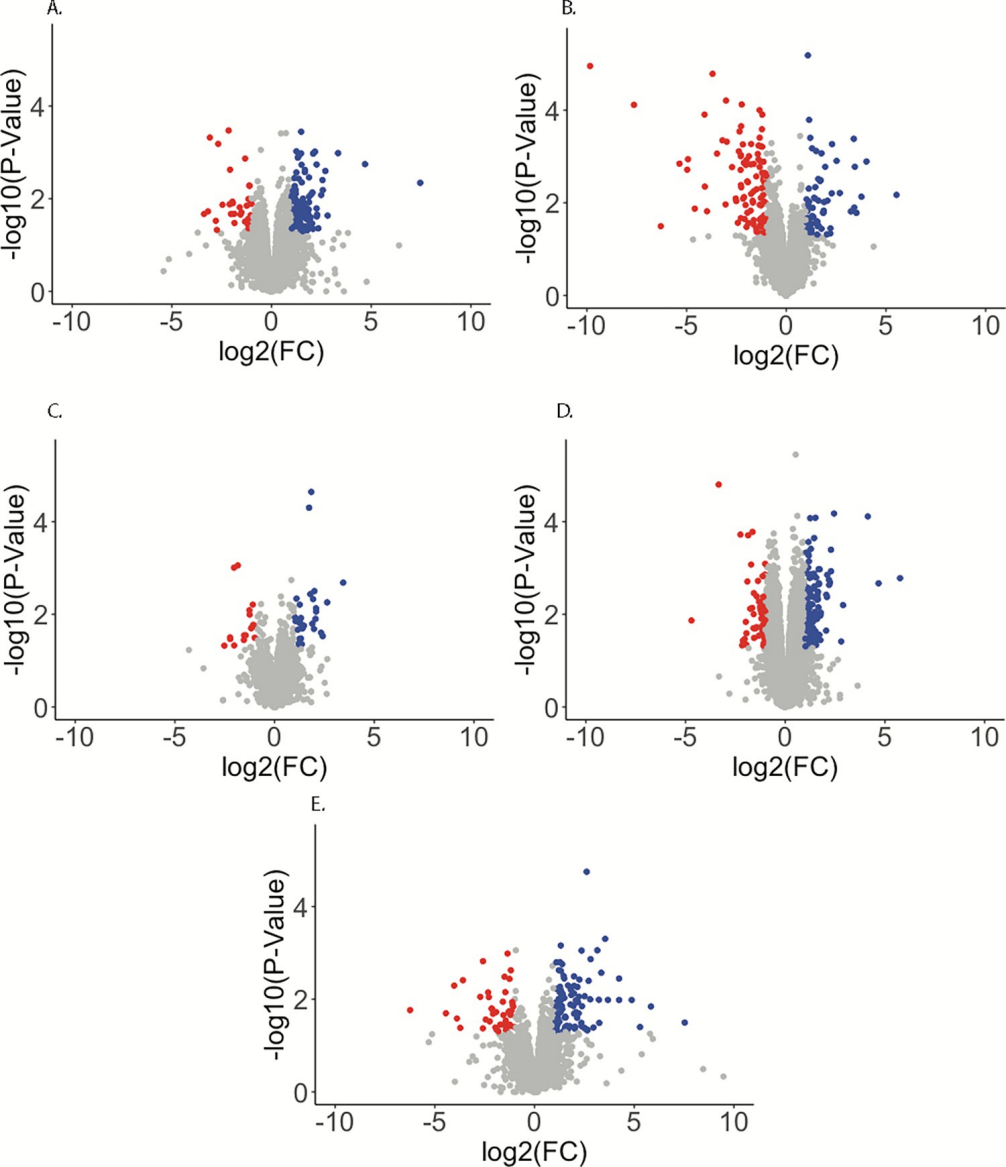
Volcano plots visualizing differential expression of *An*. *stephensi* transcripts in response to Mayaro infection. The Y-axis shows -log10 transformed P-values, and the X-axis shows log2 transformed fold change values. Red points represent transcripts depleted by more than -1 log2FC in response to infection with a FDR < 0.05, while blue points are transcripts enriched by more than 1 log2FC in response to infection with a P value < 0.05. A.—C. are transcripts regulated in the 2 dpi, 7 dpi, and 14 dpi groupings respectively, while D. and E. are transcripts regulated in the infected treatment between 2–7 dpi and 7–14 dpi respectively.

**Fig 3:**
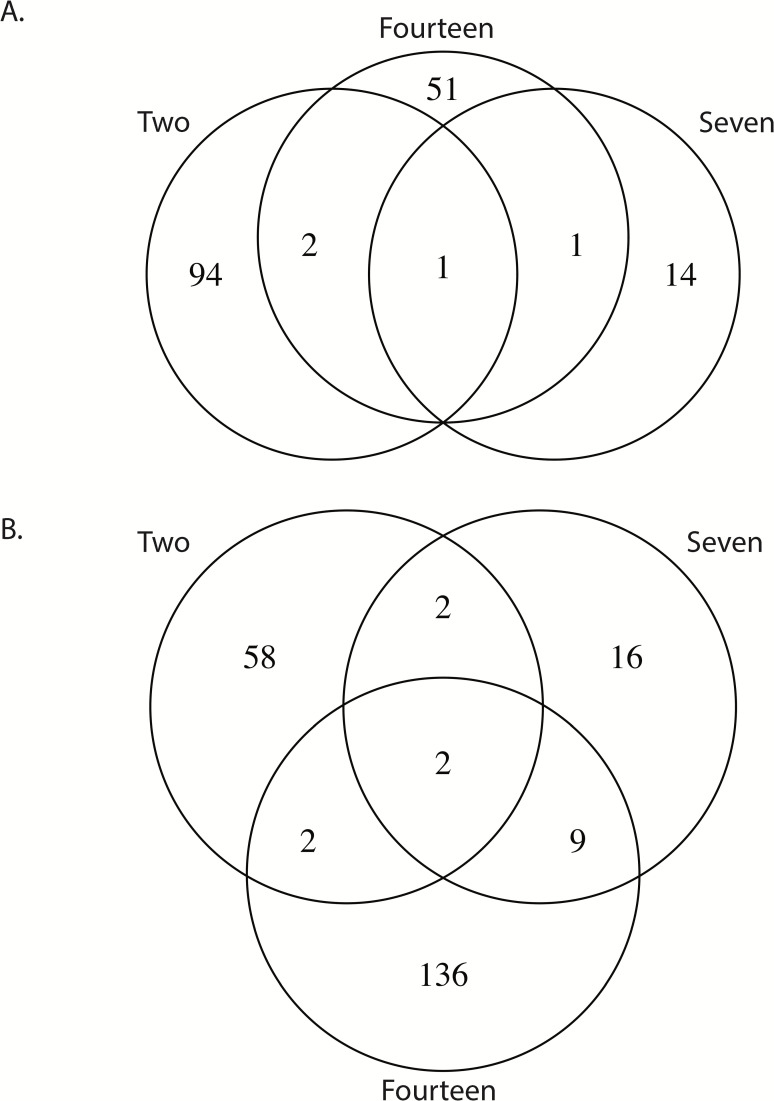
Venn diagrams representing the number of shared transcripts differentially expressed at 2, 7, and 14 dpi. A. represents the repressed transcripts and B. represents the enriched transcripts.

To determine which genes exhibit differential expression by infection status and between time points a general-linearized model (GLM) was performed on filtered and normalized read counts mapping to the AsteI2 genome (S2 Table and Figs 2 and 3). Differential expression was also computed for a newer chromosome level genome assembly (NCBI BioProject Accession PRJNA629843; S3 Table), however, the results from the older assembly are discussed here as the genes in the older assembly contain better annotations than those in the newer assembly [36]. Contrasts considered in the GLM were infected compared to control at 2, 7, and 14 dpi, and differences between 2–7 dpi and 7–14 dpi for infected treatments correcting for results from control treatments between those time points. Genes were considered significantly regulated if they had a log fold-change (log2FC) value of +/- 1 and P value < 0.05.

There were 3 genes regulated in the same direction at each time point, 2 enriched (ASTEI09037 –an ortholog of drICE caspase, and ASTEI03083 –a likely alkaline phosphatase) and 1 depleted (ASTEI04716 –ortholog of lush odorant receptor) (Fig 3). The gene with the strongest response to infection at any time point was ASTEI04601 (no *Drosophila* ortholog but conserved in mosquitoes) at 2 dpi with a log2FC of -9.8 and the most enriched gene was ASTEI04639 (no specified product) at 14 dpi with log2FC pf 5.7. When considering changes between time points for the infected treatment when controlling for the response from the uninfected treatments, there were 96 positively and 44 negatively regulated genes between 2–7 dpi, and 129 upregulated and 32 downregulated genes between 7–14 dpi. Regulated transcripts for 2–7 dpi ranged from -6.2 (ASTEI08168 –serine protease ortholog in *Ae*. *albopictus*), to 7.5 (ASTEI09252 –serine type endopeptidase ortholog in many *Aedes* and *Anopheles*) log2FC in terms of magnitude of expression, and -3.4 (ASTEI10804 –Histone H2A) to 7.4 (ASTEI04639 –no specified product) log2FC for 7–14 dpi. When considering a FDR threshold as a multiple testing correction very few transcripts in any contrast can be considered significantly regulated; 3 transcripts at 2 dpi (ASTEI04601, ASTEI05497, ASTEI05732) and 2 transcripts at 14 dpi (ASTEI00644, ASTEI08604) fall below a FDR < 0.1 threshold for significance.

#### Gene ontology

**Fig 4:**
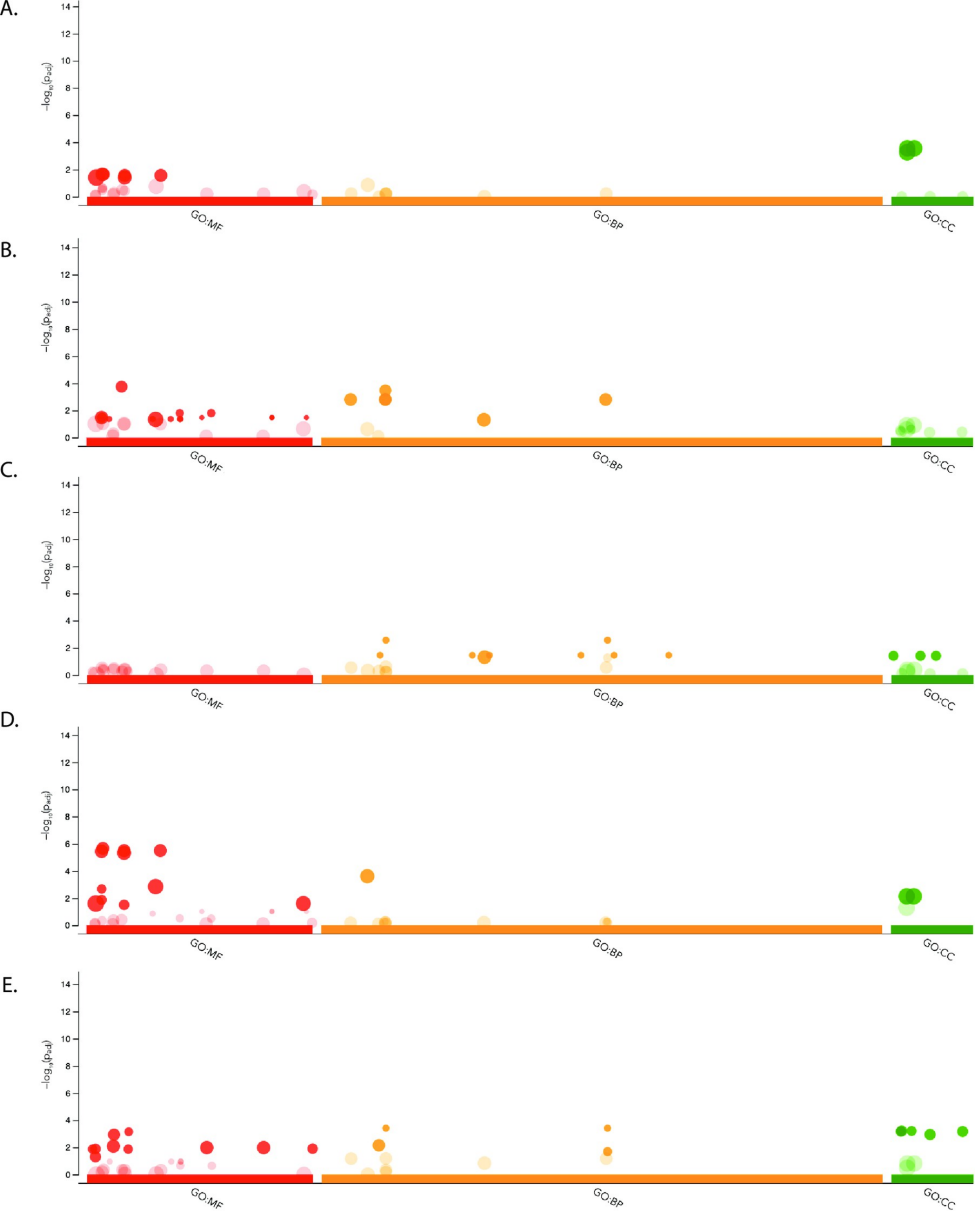
Results of g:Profiler analysis on differentially expressed transcripts in each contrast considered. Y-axis is -log10 transformed P values, and X-axis are GO categories for molecular function (MF), biological process (BP), and cellular component (CC) with the number of significant categories with FDR < 0.05 in parenthesis next to each category. Points that are transparent are those that are not significant with FDR > 0.05. Displayed are 2 dpi (A.), 7 dpi (B.), 14 dpi (C.), 2 to 7 dpi (D.), and 7 to 14 dpi (E.).

A gene ontology (GO) over-representation analysis was performed using g:Profiler on gene IDs which were significantly enriched or depleted in any considered contrast in the GLM described above when using a P value cutoff of 0.05 and any overrepresented GO terms with an FDR < 0.05 were considered significant (S4 Table and Fig 4) [33]. At 2 dpi significantly regulated molecular function terms were overrepresented by peptidase activity, specifically serine type endopeptidase activity, however this overrepresentation is not observed when just considering depleted or enriched genes it is only present when considering all regulated genes at 2 dpi together. Depleted genes at 2 dpi are overrepresented by cell-cell adhesion terms in the biological function category, and components of the membrane for cellular component, enriched genes are not significantly biased for any terms. At 7 dpi molecular function terms related to odorant binding and carboxylic acid binding were overrepresented in depleted genes, and for enriched genes however with a slightly less than significant FDR of 0.06. Molecular function terms relating to dioxygenases, transferases, and oxidoreductases are overrepresented by depleted genes at 7 dpi, and different types of carbohydrate binding such as peptidoglycan binding as well as serine and cysteine type endopeptidase terms are overrepresented by enriched genes. Biological process terms related to nervous system processes, sensory perception of smell, and mannose metabolism are overrepresented by depleted genes at 7 dpi. At 14 dpi the enriched transcripts were biased for molecular function GO terms related to sensory perception, specifically perception of mechanical, light, and sound stimuli, while depleted transcripts were biased for those related to MAPK/JNK signaling cascades, apoptosis, and amino acid biosynthesis for molecular functions and peroxisome and nucleosome for cellular component terms. From 2 to 7 dpi molecular function terms related to serine protease activity were represented by both enriched and depleted genes, but primarily by enriched genes, and enriched genes were biased for cell membrane proteins for cellular component. 7 to 14 dpi had enriched genes biased for molecular functions related to ATP dependent microtubule activity and G-protein coupled receptors while depleted genes were biased for different types of amino acid binding. Biological process terms overrepresented by enriched genes between 7 and 14 dpi were related to sensory perception, and depleted genes were overrepresented for various types of metabolic and biosynthetic processes, NF-kB signaling, and the MAPK/JNK cascade.

Endopeptidases, specifically serine proteases were enriched at 7 dpi and from 2–7 dpi, which are involved in a number of biological processes such as the Toll pathway, melanization, and digestion processes, and these are activated at a time when the virus is establishing an infection in the mosquito [33]. Activation of serine proteases is not uncommon in pathogenic infection of insects, and has been identified specifically as enriched in *Ae*. *aegypti* in response to DNV and Zika virus (ZIKV) infection, and in *An*. *gambiae* and *An*. *coluzzii* in response to o’nyong’nyong virus (ONNV) infection [16–18,37]. At late stages of infection there was depletion of the autophagic and apoptotic inducing JNK and MAPK cascades in addition to repression of JAK/STAT signaling pathways through repression of MAPK signaling. Autophagy and apoptosis both have demonstrated positive impacts on alphavirus replication [33,38], suggesting another possible molecular response from the mosquito to prevent viral replication at late stages of infection.

### Small RNA

#### miRNA identification

**Fig 5:**
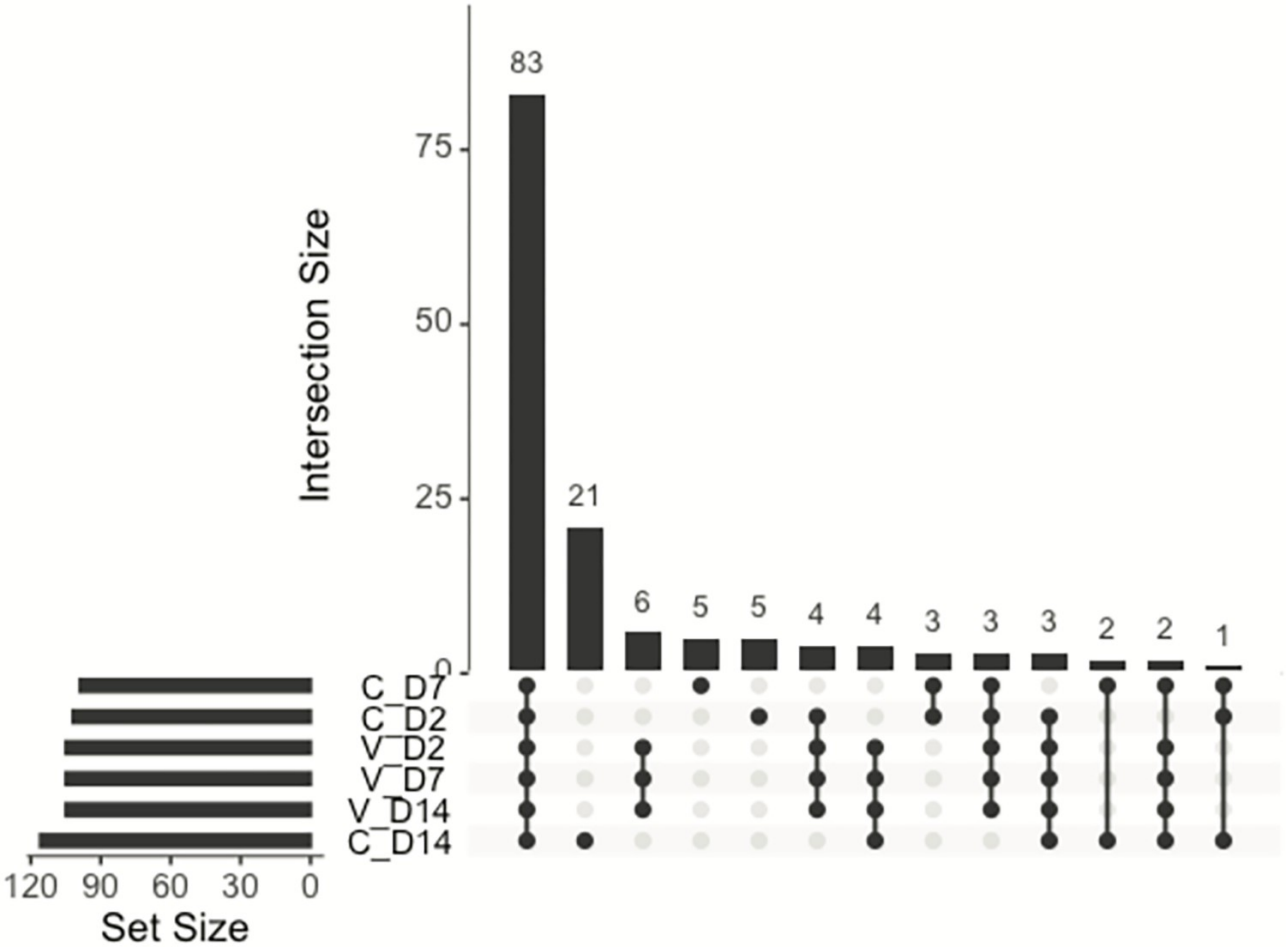
The top histogram represents the number of miRNAs shared between treatments (intersection size), and each row below the histogram represents a treatment. The lines connecting treatments below the top histogram represent treatments which share that number of miRNAs, and the histogram to the side of the treatments represents the number of miRNAs contained within each treatment. V denotes a viral sample, C denotes a control, D2, D7, and D14 represent 2, 7, and 14 dpi respectively.

**Fig 6:**
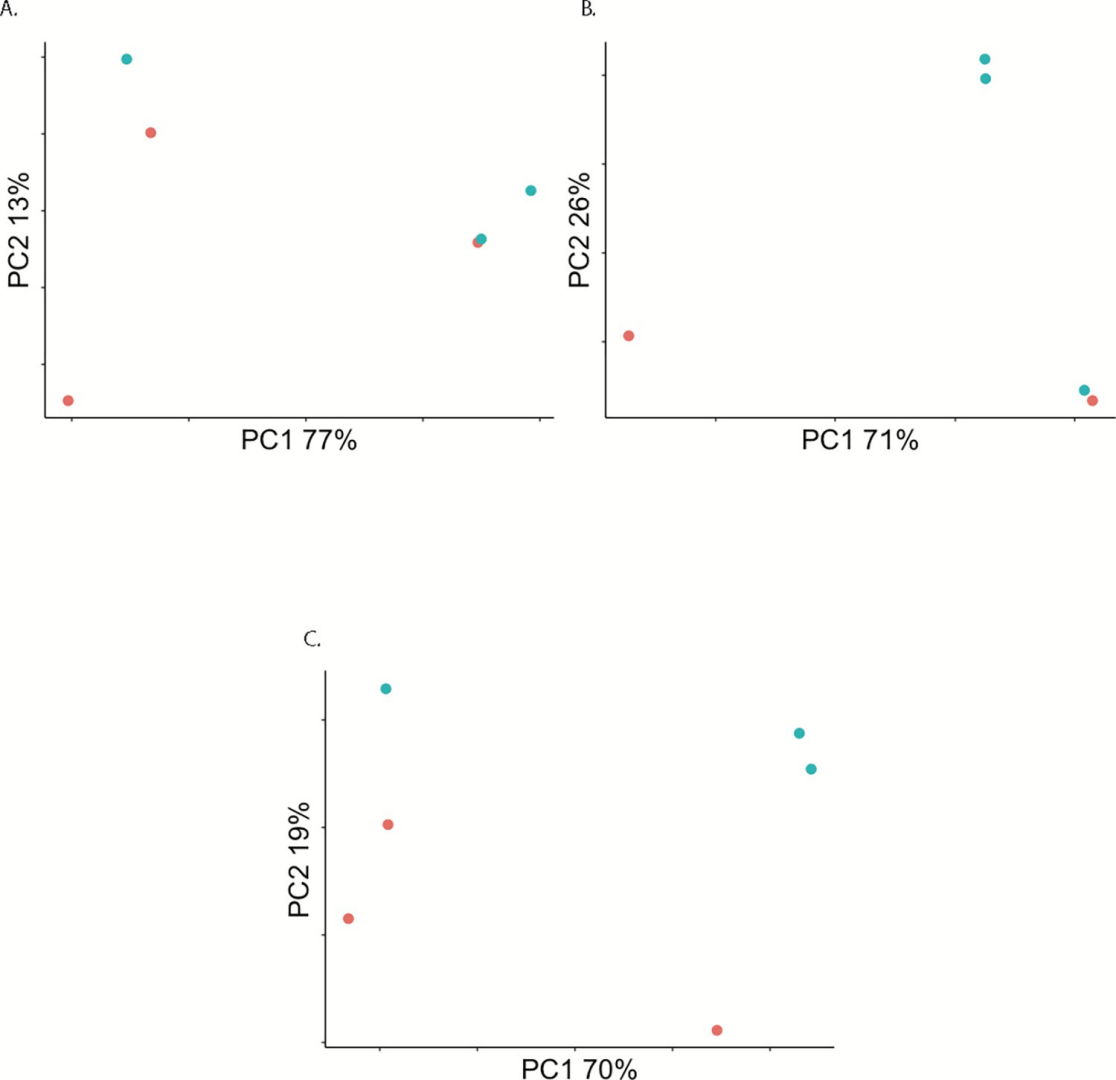
Principal Components Analysis (PCA) on read counts mapping to miRNAs identified in the AsteI2 build of the *An*. *stephensi* genome in Vectorbase. A.—C. are the 2 dpi, 7 dpi, and 14 dpi groupings respectively. In all PCAs, blue is Mayaro infected, and red is control.

We next identified novel miRNAs in the small RNA transcriptomes of the MAYV infected samples and controls using miRDeep [31]. We searched for matches in our sequencing reads to all miRNAs in the miRBase database for the species *An*. *gambiae*, *Aedes aegypti*, *Culex quinquefasciatus*, *Drosophila melanogaster*, *Bombyx mori*, *Apis mellifera*, and *Acyrthosiphon pisum*. We found matches to 73 known miRNAs, all from *An*. *gambiae*, and 78 novel miRNAs identified across all samples, with between 2.2–4.0 million reads mapping to identified miRNAs per-sample (S5 and S6 Tables). Of the 151 total miRNAs identified across all samples, 83 were present in at least one replicate per treatment (Fig 5). PCA of read counts normalized to library size for all identified miRNAs in each sample shows a correlation between infection status and grouping along the PC1/PC2 axis at each time point, however as with the PCA for transcriptome read counts there is still overlap between control and infected (Fig 6).

#### miRNA differential expression

**Fig 7:**
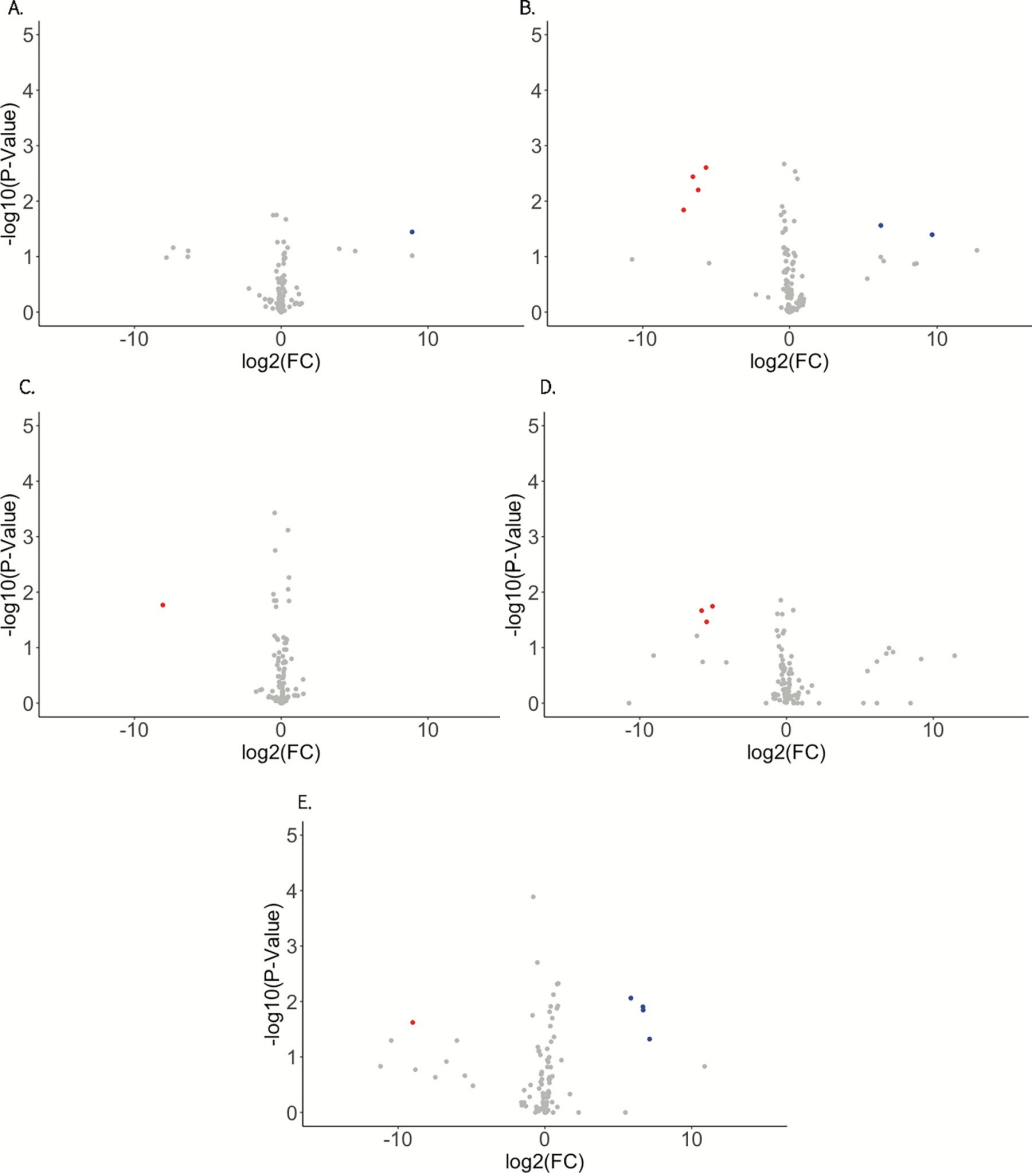
Volcano plots visualizing differential expression of identified *An*. *stephensi* miRNAs in response to Mayaro infection. The Y-axis shows -log10 transformed P-values, and the X-axis shows log2 transformed fold change values. Red points represent transcripts depleted by more than -1 log2FC in response to infection with a FDR < 0.05, while blue points are transcripts enriched by more than 1 log2FC in response to infection with a FDR < 0.05. A.—C. are the 2 dpi, 7 dpi, and 14 dpi groupings respectively, while D. and E. are miRNAs regulated in the infected treatment between 2–7 dpi and 7–14 dpi respectively.

We next identified known and novel miRNAs that were differentially expressed by infection status (Fig 7 and Tables 1 and S7). Contrasts considered in the GLM were infected against control at 2, 7, and 14 dpi, and differences between 2–7 dpi and 7–14 dpi for infected treatments relative to control. miRNAs were considered differentially expressed by having a log fold-change (log2FC) value of +/- 1 and P value < 0.05. There were a total of 8 miRNAs differentially regulated in any considered contrast, novel miRNAs as-mirNOV10, as-mirNOV16, and as-mirNOV17 as well as known miRNAs aga-miR-286b, aga-miR-2944a, aga-miR-2944b, aga-miR-307, and aga-miR-309. as-mirNOV10 was enriched at 2 dpi, as-mirNOV16 was enriched at 7 dpi, and as-mirNOV 17 was depleted at 14 dpi and between 7–14 dpi. The known miRNAs were depleted as a group at 7 dpi and in the 2–7 dpi contrast but enriched in the 7–14 dpi contrast.

**Table 1:** Differentially expressed *An*. *stephensi* miRNAs in response to Mayaro virus infection. Correlation with regulated transcript targets in the same or opposing direction in the specified contrasts are also displayed.

miRNA ID	logFC	logCPM	F	P Value	FDR	Direction	Contrast	Correlation With Significant Transcripts	Correlation Against Significant Transcripts
as-mirNOV10	8.99	4.11	6.06	0.04	0.57	POSITIVE	2 dpi		
as-mir1NOV16	6.21	2.27	6.36	0.03	0.31	POSITIVE	7 dpi		
aga-miR-309	-7.13	10.57	16.78	0.00	0.08	NEGATIVE	7 dpi	ASTEI11356	
aga-miR-286b	-7.78	11.31	11.12	0.01	0.13	NEGATIVE	7 dpi		
aga-miR-2944a	-6.23	12.45	21.04	0.00	0.07	NEGATIVE	7 dpi	ASTEI00720	
aga-miR-2944b	-6.74	10.93	13.54	0.00	0.11	NEGATIVE	7 dpi	ASTEI00720	
as-mirNOV17	-8.12	2.05	7.71	0.02	0.16	NEGATIVE	14 dpi	ASTEI10314	ASTEI09473
aga-miR-286b	-6.67	11.31	6.10	0.03	0.52	NEGATIVE	2–7 dpi		
aga-miR-2944a	-5.58	12.45	11.52	0.01	0.47	NEGATIVE	2–7 dpi		
aga-miR-2944b	-5.96	10.93	7.44	0.02	0.47	NEGATIVE	2–7 dpi		
aga-miR-309	-6.33	10.57	9.48	0.01	0.47	NEGATIVE	2–7 dpi		
aga-miR-286b	7.80	11.31	7.05	0.02	0.14	POSITIVE	7–14 dpi		
aga-miR-2944a	6.46	12.45	14.94	0.00	0.09	POSITIVE	7–14 dpi		
aga-miR-2944b	7.29	10.93	10.55	0.01	0.09	POSITIVE	7–14 dpi	ASTEI06911	
aga-miR-309	7.30	10.57	11.72	0.01	0.09	POSITIVE	7–14 dpi	ASTEI04639	
aga-miR-307	1.09	4.84	7.68	0.02	0.13	POSITIVE	7–14 dpi	ASTEI01202, ASTEI01666, ASTEI03195, ASTEI04375, ASTEI04843	
as-mirNOV17	-8.76	2.05	5.42	0.05	0.21	NEGATIVE	7–14 dpi	ASTEI10314	ASTEI06387, ASTEI08426, ASTEI11105

The miR-309/286/2944 have been found to be enriched in *An*. *gambiae* in response to blood feeding [39–40], and to be associated with Argonaute proteins post-bloodmeal [39]. When experimentally repressed aga- miR-309 was found to retard oocyte development [39], depletion in response to MAYV infection may suggest that viral replication or the host immune response sequesters resources normally requires for host oocyte development, and as a result associated miRNAs are also depleted. Experimental infections have demonstrated that for *Ae*. *aegypti*, infection with CHIKV does not impact the number of eggs laid but it does have a detrimental impact on the viability of the eggs produced, however this is not observed with ZIKV [41,42]. Infection of *Culex tarsalis* with West Nile Virus demonstrates a decrease in fecundity as measured by egg raft size and number of eggs laid [43].

#### miRNA target prediction

We next identified putative targets in the *An*. *stephensi* genome for all known and novel miRNAs identified in all samples [32]. For each of the identified miRNAs we found an average 537 potential annotated targets within the Astel2 genome (S8 Table). Targets for significantly regulated miRNAs were loaded into g:Profiler and any overrepresented GO terms with an FDR < 0.05 were considered significant (S9 Table) [33]. We also performed Kendall rank correlation on logFC values of significantly regulated miRNAs against significantly regulated transcripts to determine what relationships existed between the two (Tables 1 and S10). FDR on Kendall rank correlation was also performed, but no correlations could be considered significant with FDR < 0.05 due to the large number of tests necessary to investigate correlations between all significantly regulated miRNAs and transcripts.

No overrepresented GO terms were identified by the genomic targets of as-mirNOV-10 or aga-miR-307. as-mirNOV-16 was significantly enriched in response to infection at 7 dpi and the only GO terms overrepresented by the predicted genomic targets of this miRNA are associated with protein binding. as-mirNOV17 was depleted at 14 dpi and between 7–14 dpi and has GO terms related to transmembrane ion channels overrepresented by its genomic targets. aga-mir-2944a and aga-mir-2944b were both depleted at 7 dpi and between 2–7 dpi but enriched between 7–14 dpi and both have GO terms primarily associated with intracellular signaling and various binding functions, and aga-mir-2944b also appears to be involved with lipid localization and transport. aga-mir-286b and aga-miR-309 were both also depleted at 7 dpi and between 2 and 7 dpi, and were enriched between 7 and 14 dpi; aga-mir-286b only had acetylglucosaminyltransferase activity overrepresented by its genomic targets, and aga-miR-309 had terms related to calcium ion transport, actin filament binding, and catalytic activity overrepresented by its genomic targets.

The targets of novel miRNA as-mirNOV-17 are significantly correlated with 1 enriched (ASTEI09473 –cytochrome P450 ortholog in *Aedes* and *Anopheles*) and 1 depleted (ASTEI10314 –no specified product) transcript target at 14 dpi and 3 enriched (ASTEI06387 –no specified product, ASTEI11105 –no specified product, and ASTEI08426 –TPR domain containing protein) and 1 depleted (ASTEI10314) transcript target between 7 and 14 dpi, when as-mirNOV-17 was significantly repressed in response to MAYV infection. aga-miR-309 is significantly correlated with 1 depleted transcript target (ASTEI11356 –lysosomal alpha-mannosidase ortholog in *Aedes* and *Anopheles*) at 7 dpi and 1 enriched transcript target (ASTEI04639 –no specified product) between 7 and 14 dpi when it was depleted and enriched respectively. aga-miR-2944a and aga-miR-2944b are significantly correlated with 1 depleted transcript target (ASTEI00720 –ortholog of class B scavenger receptor in *Aedes* and *Anopheles*) at 7 dpi when they are depleted, and aga-miR-2944b is significantly correlated with 1 enriched transcript target (ASTEI06911—beta-hexosaminidase ortholog in *Aedes* and *Anopheles*) between 7 and 14 dpi when it is enriched. aga-miR-207 is correlated with 5 enriched transcript targets (ASTEI01202 –homeobox domain containing protein ortholog in *Aedes* and *Anopheles*, ASTEI01666 –SBF2 domain containing protein, ASTEI03195 –no specified product, ASTEI04375 –homeobox domain containing protein ortholog in *Aedes* and *Anopheles*, and ASTEI04843 –sodium/potassium/calcium exchanger ortholog in *Aedes* and *Anopheles*) between 7 and 14 dpi when it also is enriched.

While some miRNAs showed correlation with targets in the opposite direction of their regulation consistent with their acting as effector molecules for RNAi, the majority of correlation relationships showed regulation of targets in the same direction as the miRNA [38]. Recent studies have demonstrated that through targeting of promotor elements miRNAs can have a positive impact on gene transcription, so this could explain the phenomenon happening where miRNA targets are enriched when the miRNAs themselves are also enriched [44–45].

#### piRNA identification

**Fig 8:**
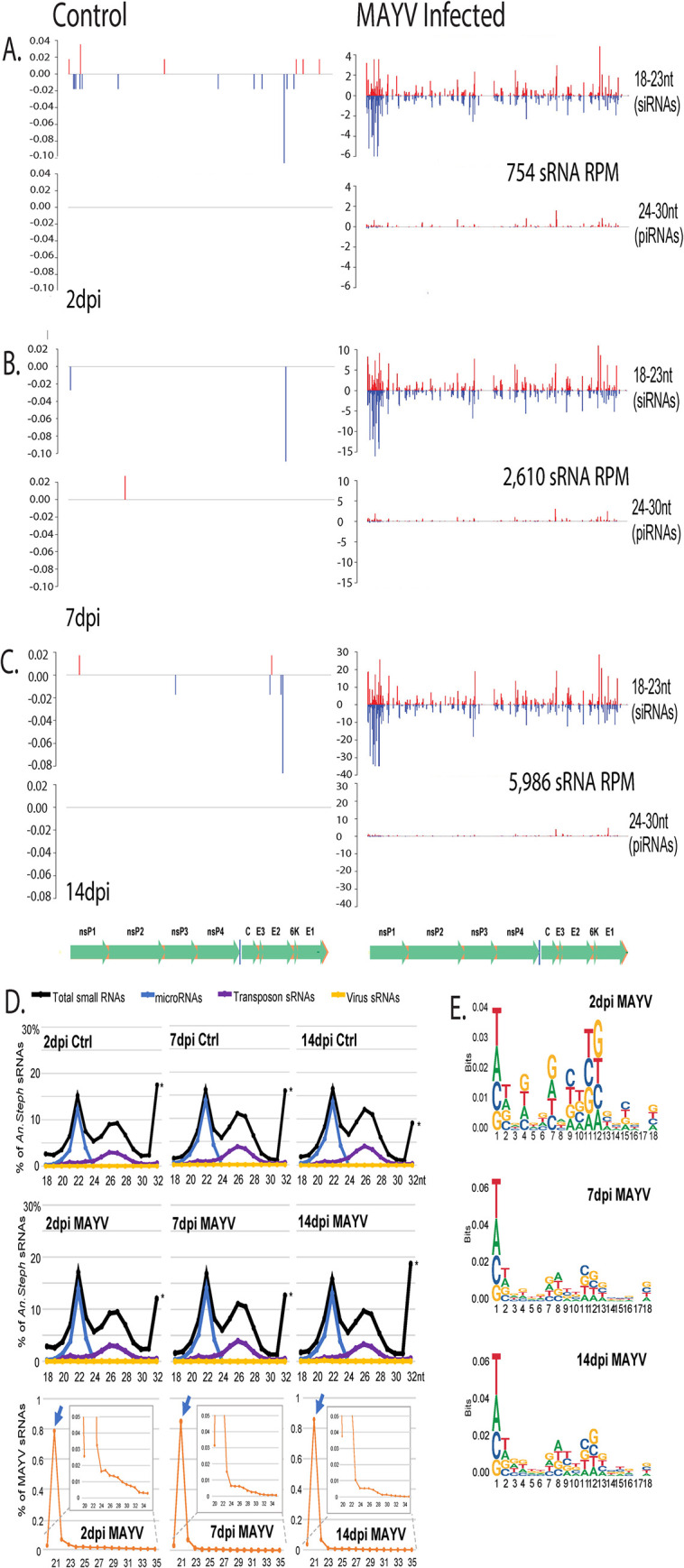
Histograms demonstrating read depth across the Mayaro virus (MAYV) genome for reads with a siRNA size profile (18-23nt) and a piRNA size profile (24–35 nt). Y-axis is read depth in reads per million, and X-axis is position in viral genome. Blue color reads map to the negative strand while red color reads map to the positive strand. A.—C.: 2, 7 and 14 dpi respectively of control (left) and infected (right) time points. At the bottom is a map of the MAYV genome. D. Length distributions of the total small RNAs in *An*. *stephensi* days after the blood meal with control versus MAYV-infected blood. The asterisks mark an artificial RNA size marker present in these libraries. Bottom row are the length distributions of MAYV-specific small RNAs, with the inset showing the progressive decrease of piRNA-long reads and the arrows noting the increase in the siRNA proportion. E. Sequence logos showing the nucleotide base compositions of the MAYV-specific small RNAs, which are mostly siRNAs, over the different days post blood meal.

We identified putative piRNAs in the trimmed small RNA datasets for each sample by isolating all 24–35 nt reads, removing those that were identified positively as miRNAs, and mapping the remaining reads to the Mayaro Virus NC_003417.1 genome using the Bowtie sequence aligner, all of which was performed using the MSRG pipeline [26–27,34,46]. There was no explicit evidence for viral piRNA expression in infected samples, and the proportion of piRNA-size small RNAs, which could contain siRNAs, mapping to the viral genome remained consistent across time points with no particular peaks or hotspots identified across the viral genome (Fig 8) [47].

Virus-derived piRNA-like small RNAs, have been identified in insects and insect cells infected with Flaviviruses, Bunyaviruses and Alphaviruses. Knockdown of the piRNA pathway proteins leads to enhanced replication of arboviruses in mosquito cells, suggesting their potential antiviral properties in mosquitoes and *Culicoides* [48–53]. For example, knockdown of Piwi-4 in *Ae*. *aegypti* Aag2 cell line increased replication of SFV, and silencing of Ago3 and Piwi-5 led to significantly reduced production of piRNAs against Sindbis virus (SV) [47,50].

#### siRNA identification

We identified putative siRNAs in the trimmed small RNA datasets for each sample by isolating all 18–24 nt reads, removing those that were identified positively as miRNAs, and mapping the remaining reads to the Mayaro Virus NC_003417.1 genome using the Bowtie sequence aligner, all of which was performed using the MSRG pipeline [26,27,34,46]. There was minimal alignment of potential siRNAs to the MAYV genome in the control samples, but in the infected samples there was significant siRNA alignment across the genome (Fig 8). There was no particular bias for positive of negative strand mapping of the siRNAs to the MAYV genome at any time point, however, there were two peaks for siRNA alignment, one at nonstructural protein 1 and another at envelope protein 1 and the number of siRNA reads mapping to the viral genome increased from 2 to 14 dpi. Because the main response is siRNAs, the only meaningful base composition bias is a preference for T/U as the first nucleotide base, which is the same preferred base of microRNAs and piRNAs that all Argonaute proteins evolved to bind [54]. The increase in siRNA reads mapping to the viral genome is suggestive of an increase in viral genome copies, degradation of viral genomes accumulating over time, or that tissues infected at later stages of infection are more prone to an siRNA response.

The siRNA pathway is thought to be the main antiviral component of immunity in insects at the cellular level (See [44,55]. Functional studies in *Aedes* mosquitoes have implicated the siRNA pathway as integral to the antiviral response in the midgut stage of infection as well as at later systemic stages of infection for DNV and SV [55–60]. Studies of the siRNA response in *An*. *gambiae* have shown that ONNV does stimulate a siRNA response at early midgut stages of infection but it is not antivirial in the midgut, it is however antivirial at later stages of infection in the systemic compartments [23]. Our results differ from the findings from *An*. *gambiae* and ONNV in that the siRNA response is detectable at early stages of infection and is persistent until later stages.

### Conclusions

The transcriptomic profiles suggest that MAYV activates protease expression, specifically serine proteases at early and mid-stages of infection. This protease activation could signal activation of Toll, melanization, digestion, or a number of other pathways following ingestion of a MAYV infectious bloodmeal [61]. At later stages of infection there appears to be a repression of JNK and MAPK signaling cascades, potentially impacting autophagic and apoptotic processes as a way to limit MAYV replication. We observed miRNAs elicited in response to infection and identified some significant correlations with transcripts identified as regulated in response to infection. Overall total and bulk small RNAs, including miRNAs and transposon-targeting sRNAs, are not altered much in *An*. *stephensi* between control and MAYV-infected bloodmeal. *An*. *stephensi* does have a small RNA response to MAYV, first with a rapid response at 2 dpi generating mostly viral siRNAs and some small proportion of piRNAs, and with time this proportion becomes even more greatly skewed to siRNAs.

## Supporting information

S1 Table Information related to infection of *An*. *stephensi* with Mayaro virus. Includes number of mosquitoes in each treatment and time point and associated mortality, nanodrop readings for all RNA extractions collected, pooling scheme for sequencing of mRNA and small RNA, and qPCR data from each sample using primers specific for Mayaro virus strain BeAn to confirm infection status.
(XLSX)

S2 Table Differentially expressed transcripts from the *An*. *stephensi* AsteI2 genome. (XLSX)

S3 Table Differential expression results from the *An*. *stephensi* PRJNA629843 genome. (XLSX)

S4 Table GO term overrepresentation for differentially regulated transcripts. (XLSX)

S5 Table Read counts mapping to the identified *An*. *stephensi* miRNAs in each small RNA sample sequenced, both raw counts and counts normalized to library size (reads per million, RPM) are presented. (CSV)

S6 Table All known and novel mature and precursor miRNA sequences identified. (CSV)

S7 Table Differential expression of *An*. *stephensi* miRNAs. (XLSX)

S8 Table Genomic targets from the *An*. *stephensi* AsteI2 genome for all identified miRNAs. (XLSX)

S9 Table Overrepresented GO terms represented by targets of significantly regulated miRNAs. (XLSX)

S10 Table Kendall rank correlation on logFC values of significantly regulated miRNAs against significantly regulated transcripts. (XLSX)
